# Characterization of the Human Folate Receptor Alpha Via Novel Antibody-Based Probes

**DOI:** 10.18632/oncotarget.412

**Published:** 2011-12-27

**Authors:** Daniel J. O'Shannessy, Elizabeth B. Somers, Earl Albone, Xin Cheng, Young Chul Park, Brian E. Tomkowicz, Yoshitomo Hamuro, Thomas O. Kohl, Tracy M. Forsyth, Robert Smale, Yao-Shi Fu, Nicholas C. Nicolaides

**Affiliations:** ^1^ Morphotek Inc., 210 Welsh Pool Road, Exton, PA 19342, USA; ^2^ Centocor Inc. Malvern, PA, USA; ^3^ ExSAR Corporation, Monmouth Junction, NJ, USA; ^4^ Rockland Immunochemicals, Inc., Gilbertsville, PA, USA; ^5^ Laboratory Corporation of America, Los Angeles, CA, USA

**Keywords:** Folate receptor alpha, cancer, diagnostics, monoclonal antibody, protein structure, 26B3

## Abstract

Folate receptor alpha (FRA) is a cell surface protein whose aberrant expression in malignant cells has resulted in its pursuit as a therapeutic target and marker for diagnosis of cancer. The development of immune-based reagents that can reproducibly detect FRA from patient tissue processed by varying methods has been difficult due to the complex post-translational structure of the protein whereby most reagents developed to date are highly structure-sensitive and have resulted in equivocal expression results across independent studies. The aim of the present study was to generate novel monoclonal antibodies (mAbs) using modified full length FRA protein as immunogen in order to develop a panel of mAbs to various, non-overlapping epitopes that may serve as diagnostic reagents able to robustly detect FRA-positive disease. Here we report the development of a panel of FRA-specific mAbs that are able to specifically detect FRA using an array of diagnostic platforms and methods. In addition, the methods used to develop these mAbs and their diverse binding properties provide additional information on the three dimensional structure of FRA in its native cell surface configuration.

## INTRODUCTION

Folate receptor alpha (FRA) is a glycosylphosphatidylinositol (GPI) anchored cell surface glycoprotein that is able to bind free folate with high affinity [1]. It is a member of a family of proteins whose function is the management of folate transport in cells. FRA is distinct from the more ubiquitously expressed reduced folate carrier (RFC) or the intestine-localized proton coupled folate transporter (PCFT) proteins, as these are both membrane-spanning receptors that facilitate *bidirectional* transport of reduced folate across the plasma and endosomal membranes [2].

FRA is a member of a family of folate binding receptors that have diverse structural identities but mediate *unidirectional* transport of folates into cells. Four isoforms of this receptor family have been identified and are designated as folate receptor alpha (FRA), beta (FRB), delta (FRD) and gamma (FRG), respectively. The FRA and FRB isoforms are both GPI-anchored proteins with two N-glycosylation sites and have high affinity (K_D_ ~1 nM) for folic acid/vitamin B9 [3]. These two family members share the highest identity among this protein family. It is postulated that these receptors function as folate scavengers when folate supply is low or rapid cell growth requires elevated uptake of folate for methylation reactions including DNA biosynthesis. The expression of FRA and FRB are distinct in normal and malignant tissues. In normal tissue, FRA is mainly expressed on the apical surface of a subset of polarized epithelial cells whereas its aberrant expression has been prominently correlated with malignancies of epithelial origin [4]. FRB has been found to be mostly limited to hematopoietic cells of the myelogenous lineage [5]. Table 1 compares and contrasts the properties of the ubiquitous RFC and the tissue-specific FRA and FRB proteins.

**Table 1:** Selected Properties of the FRA, FRB and RFC proteins

Characteristic	FRA	FRB	RFC
**Protein Structure**	Single chain/GPI linked	Single chain/GPI linked	Single unit/transmembrane
**Molecular weight [25, 26]**	38kDa	34kDa	60kDa
**Cellular Distribution**	Restricted to epithelial cells	Restricted to lymphoid cells	Ubiquitous
**Folate binding properties:**			
**Folic acid**	Yes	Yes	No
**5-methyltetrahydrofolate**	Yes	Yes	Yes
**Anti-folates**	Yes	Yes	Yes
**Binding affinity for folic acid [27]**	1 nM	1nM	200-400μM
**Binding affinity for 5-mTHF and other reduced folates [25]**	1–10 nM	1-10nM	1–10μM

FRA is the most widely studied folate receptor from this family due to its restricted expression in normal tissues and high expression patterns in various epithelial derived tumors [6]. FRA expression and association with malignant cells was further supported by tumor antigen discovery studies using human tumor cells in immune-competent mice in an unbiased effort to identify cell surface tumor antigens via humoral immune responses to proteins expressed by tumor cells [7]. These efforts led to the discovery of an antibody called LK26, whose tumor specific binding to fresh frozen malignant tissues using immunohistochemistry (IHC) led to the pursuit and isolation of the antigen that in turn was identified to be FRA. A humanized version of the LK26 antibody was subsequently generated and found to have identical binding properties to the LK26 precursor. This humanized form is called MORAb-003 as well as the generic name, farletuzumab.

Since its discovery, several tumor association studies have been conducted using FRA specific antibodies that are able to recognize the receptor in the native but not denatured state, including the LK26 mAb. Protein expression studies have demonstrated FRA to be highly and uniformly present in non-mucinous carcinomas of the ovary and endometrium, while its expression was also found in other epithelial tumors including non-small cell lung adenocarcinoma (NSCLC), clear cell renal carcinoma, primary and metastatic colorectal carcinoma and breast carcinoma at lesser frequencies [8, 9, 10, 11]. In ovarian cancer, several reports have found that FRA expression increases with tumor stage [12], and is associated with decreased survival [13].

The FRA pathway has been deemed to be oncogenic in nature, based on its high correlation with malignant tissues in patients as well as from experimental studies conducted by independent laboratories. *In vitro* studies have found that ectopic over-expression of FRA in normal cells can result in cellular transformation that can be reversed by suppressing its expression. Other studies have shown that inhibition of FRA expression in naturally expressing FRA positive tumor cell lines also suppresses cellular proliferation [14, 15, 16]. *In vivo* studies using human FRA-expressing tumor xenografts in mice have confirmed the ability to suppress tumor growth using anti-FRA mAbs that can perturb its biological activity [17]. The mechanism by which FRA supports tumorigenesis is still unclear. While it is possible that recruitment of more folate to cells is a mechanism for tumorigenesis, it is important to consider other mechanisms as well. Isolation of FRA from the membrane of ovarian cancer cells has demonstrated it to be associated with several signal transduction molecules that in turn may be involved in signaling for enhanced growth [18]. Efforts are underway to further elucidate the mechanism by which FRA supports tumor cell growth. Despite the lack of understanding of the entire mechanism by which FRA supports cellular transformation and tumor cell growth, the association of the protein with specific cancer subtypes, as well as the independent experimental studies described above, strongly support a role for FRA and its pathway in cancer as a bonafide candidate for targeted cancer therapy [19].

Significant activity in the drug development arena is focused on targeting FRA based on its' highly tumor restricted expression profile. Several agents are currently in late-stage clinical development that target FRA itself, or use the folate binding activity of FRA to deliver folate-conjugated toxins. While FRA expression has been reported to be highly correlated with certain cancers such as ovarian and endometrial, patient pre-selection in cancers where the frequency of expression is less than ubiquitous is important to determine those who may benefit from FRA mediated therapy [20, 21, 22]. Unfortunately, due to the complex secondary structure of the FRA protein, the development of reagents that can detect the protein with reproducible specificity and high sensitivity is required to support the accurate diagnosis of patients with FRA positive cancers. In light of the differences in reagents and methods used for tissue procurement, varying frequencies of expression of FRA in one of the most homogenously expressing cancers, ovarian carcinoma, have been reported, further supporting the need for development of more robust diagnostic reagents [23, 24]. Given these needs, we embarked on a program to develop specific reagents that may prove useful for the sensitive and specific detection of FRA in tissue or in biological fluids such as serum, plasma, urine or sputum. Here we describe the development of novel, high-affinity mAbs that are FRA specific and can be applied to multiple diagnostic platforms. Moreover, due to the highly complex structure of the FRA protein, the analysis of the receptor using the reagents described herein has led to the further refinement of the three dimensional architecture of this protein. These efforts may aid in elucidating the function of how FRA supports tumorigenesis.

## RESULTS

In the present work, two different immunogens were used in an attempt to generate FRA specific mAbs; a full length recombinant human FRA (rFRA) and a full length denatured FRA (dFRA) obtained by urea denaturation followed by reduction and alkylation. FRA is a disulfide linked protein, containing seven disulfides and, based on similarities to the chicken riboflavin-binding protein (cRBP) and the X-ray structure thereof, is thought to be a highly compact structure [25]. Based on this structure, it is expected that only a limited number of possible epitopes are present in the native protein that may be recognized by the immune system. In order to expand the number of possible epitopes, we generated the dFRA with the expectation that it would be less structurally constrained and more linear in nature. This immunogen would potentially have an increase in the number of distinct residues that could yield a broad panel of novel antibodies that may be useful in the robust and reproducible detection of FRA in tissues processed by a variety of methods, other biological samples such as serum, as well as being useful in providing additional information about the three dimensional structure of FRA.

The immunizations using rFRA and the dFRA produced strikingly different responses whereby a total of 7 and 62 parental anti-FRA producing hybridoma lines were generated, respectively. These differences are most likely due to the immunogen structure since the procedure for immunization of both antigens, and screening of clones, were conducted using identical methods. These data imply that disruption of the tertiary structure of FRA, by reduction of the disulfides followed by alkylation of the cysteines, significantly improves its immunogenicity and suggests that this procedure might be useful for developing broad panels of mAbs to other high disulfide-rich proteins as well. One possible reason for the low number of anti-FRA antibodies derived from rFRA immunization is the high degree of homology that may exist between the native human and mouse proteins that resulted in tolerance to the rFRA protein in the murine host. Conversely, the dFRA may have more exposed epitopes and unmasked non-conserved sequences/domains between the two species. Analysis of the mouse and human FRA proteins demonstrates that an 81% identity exists at the primary amino acid sequence level, whereas folding and external surface exposure of conserved regions may result in higher similarities between the two species [28]. Further characterization of the antibodies generated using both immunogens found that most of those obtained from the rFRA immunizations were of relatively low affinity as determined by BIAcore analysis, further supporting the hypothesis that the native human and mouse proteins share a high degree of homology on the external surfaces of the native proteins. Of the seven rFRA-derived antibodies, only one (9F3), proved to be suitable for FRA recognition using the battery of assays described below while the majority of dFRA-derived antibodies had high affinity and robust FRA binding (Table 2). Interesting is the fact that the EIA assay used to screen for anti-FRA antibodies from both immunogens employed the non-denatured rFRA thereby demonstrating the ability of these antibodies to presumably recognize epitopes present in FRA in the native state. To better characterize these antibodies, twenty of the best binders as scored by EIA were further analyzed for FRA binding using a variety of assays including FACS and IHC of positive and negative FRA expressing tissues.

**Table 2:** Summary Characteristics of Selected mAbs

Antibody	Immunogen Used to Develop mAb	IgG isotype	IHC Reactivity FFPE (+/-)	FACS Analysis+ on FR Expressing Cell Types			Reduced WB Reactivity (+/-)
				FRA	FRB	FRD	
9F3	rFRA	IgG2a	(−)	760	6	5	(−)
24F12	dFRA	IgG1	NT*	777	6	6	(−)
26B3	dFRA	IgG1	(+)	854	6	6	(−)
19D4	dFRA	IgG2a	(+)	1130	NT	NT	(−)

* NT = Not Tested; +values represent fluorescence intensity

rFRA = full length recombinant human folate receptor alpha

dFRA = full length denatured, reduced and alkylated human folate receptor alpha

**Figure 1:**
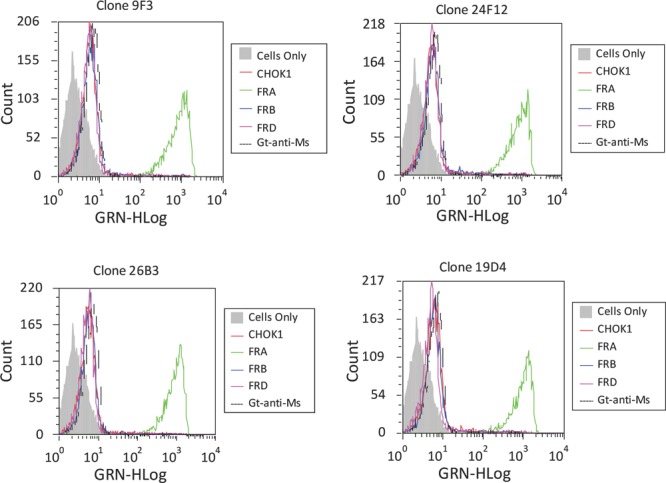
Fluorescence Activated Cell Sorting (FACS) Antibodies were tested for their ability to detect FRA on recombinant CHO cells expressing human FRA, FRB or FRD. Twenty of the 69 mAbs developed here were able to specifically recognize FRA and not the other folate receptor orthologs or negative controls. Shown is a representative analysis of lead antibodies and their robust FRA-specific binding. We then tested the ability of these antibodies to cross react to epitopes or with the previously reported anti-FRA mAb MORAb-003 which is being pursued in clinical development for the treatment of ovarian cancer [20]. Very few mAbs were found to compete for binding. Of particular note was the cross reactivity of 24F12 with 26B3 and 19D4 with MORAb-003 (not shown).

FACS analysis of the lead antibodies using recombinant CHO cell lines expressing the human FRA, or the orthologs FRB and FRD, was conducted to test for binding specificity. Results of these studies found that all 20 antibodies were able to specifically bind CHO-FRA but not cells expressing the FRB or FRD orthologs. Results depicting the FACS binding for a subset of these antibodies are shown in Figure 1. Competition studies were also performed on these cells using various combinations of antibodies to determine if any of the mAbs competed with each other for FRA binding. As expected a number of antibodies appeared to compete with each other for FRA binding and only one competitive antibody from each group was further assessed for binding in epitope mapping studies described below. The LK26 antibody that was originally developed using a whole tumor immunization protocol was used as a positive control to confirm FRA expression and mAb specific binding [7]. Competition studies using the new mAbs generated here found that only one, 19D4, was able to compete for binding of LK26, suggesting overlapping epitopes or perturbation of the LK26 epitope by 19D4.

**Figure 2:**
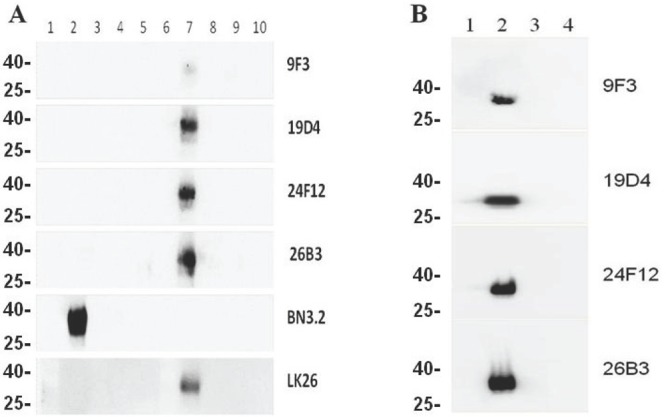
Western blotting of mAbs to reduced and non-reduced FR isoforms Purified recombinant (panel A) and whole cell lysates (panel B) from CHO cells expressing FRA or FR homologs FRB, FRD or FRG were run on SDS-PAGE gels. Proteins were prepared in sample buffer with or without reducing agents. Panel A, lane 1, molecular weight markers, lanes 2-5. 0.5μg reduced FRA, FRB, FRG, and FRD, respectively; lane 6, blank; lanes 7-10, 0.5μg non-reduced FRA, FRB, FRG, and FRD, respectively. The positive band represents the only reactive species in each lane and corresponds to a molecular weight of ~38kDa. Panel B, lane 1 molecular weight markers, lane 2 CHO-FRA, lane 3, CHO-FRB, lane 4 CHO-FRD whole cell lysates prepared in sample buffer without reducing agents on a SDS-PAGE gel. Each panel is probed with the designated anti-FRA mAb labeled on the right. The molecular weights for FR are: FRA ~38kDA; FRB ~30kDa; FRG ~28kDa; FRD ~26kDa.

We next tested the ability of the antibodies to bind rFRA or FRA from recombinant CHO cell lysates expressing FRA and other orthologs ectopically via western blots under reduced and non-reduced conditions. Figure 2 shows a representative analysis of the ability of these antibodies to recognize FRA under both conditions. The FRB, FRD and FRG orthologs were included as controls to test for binding specificity. The LK26 and BN.3 antibodies that recognize FRA under denatured and non-reduced or reduced only conditions were used as positive controls, respectively [7, 29]. Nineteen of the twenty mAbs tested were able to robustly detect FRA when run under denatured and non-reduced conditions while no cross reactivity to FRB, FRD or FRG was observed for any antibody. This result is somewhat surprising in light of the fact that all of the antibodies except for 9F3, which only weakly recognized non-reduced FRA under denatured conditions, were derived from immunizations using the dFRA preparation but may reflect the stringent screening process employed in the present work. These data further demonstrate that while most of these antibodies recognize linear epitopes (as determined by reactivity on denatured non-reduced western blots) a subset of them recognize conformational or constrained epitopes as determined by their inability to react with FRA on reduced western blots. This data would suggest conformation-specific epitopes for several of these antibodies and their epitopes could provide insight into the three dimensional structure of the native FRA protein. Indeed, H/D exchange epitope mapping of several of these antibodies suggest non-linear epitopes and/or epitopes that are flanked by cysteines that likely provide secondary structure (see below).

**Figure 3:**
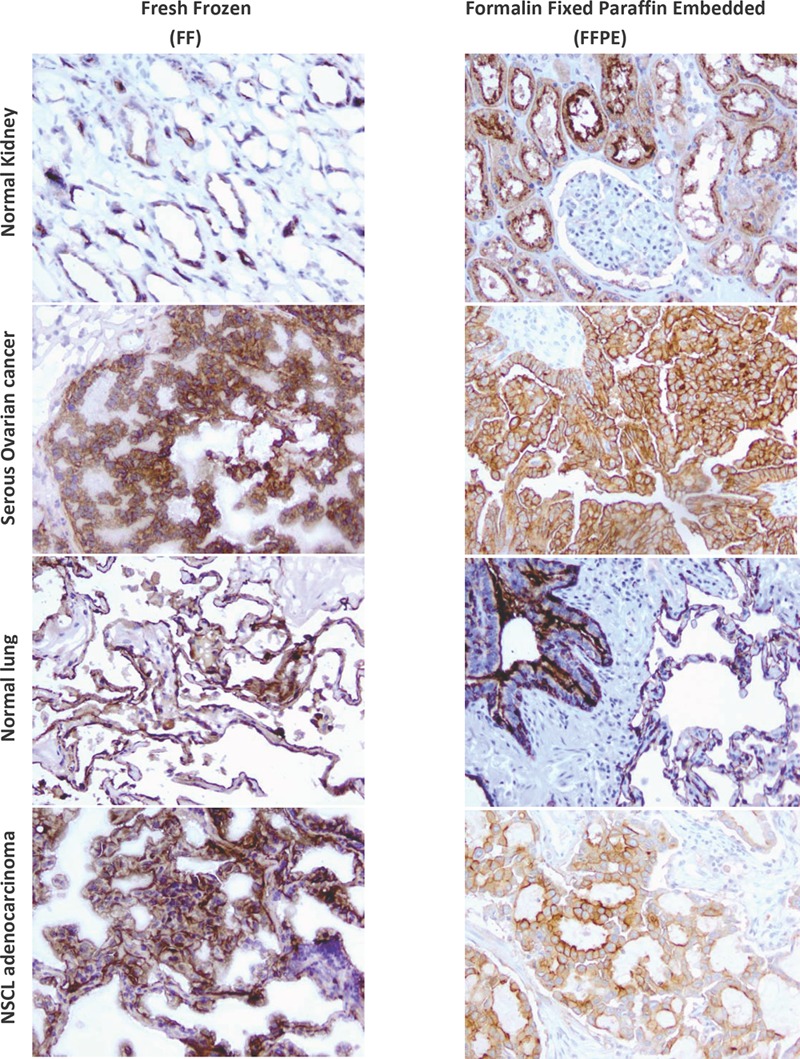
IHC of mAbs on human tissues mAbs were used to assess the ability to detect FRA in tissues prepared via FFPE or FF methods. Shown is a representative analysis of anti-FRA mAbs developed here that demonstrate specific staining of FRA positive tissues prepared using various methods. mAb 26B3 showed the most robust staining for FRA in IHC and is presented above. Shown is the ability of 26B3 to detect FRA in normal tissues and previously identified FRA-positive cancers (serous ovarian cancer and NSCLC adenocarcinoma) prepared using formalin fixed paraffin embedded (FFPE, right column) and fresh frozen (FF, left column) tissue. As shown, mAb 26B3 is able to robustly recognize antigen in both tissue preparations. Slides shown at 20X magnification.

When performing immunohistochemistry (IHC) to probe for antigen expression in FFPE (Formalin Fixed Paraffin Embedded) processed tissue samples, antibodies typically recognize a linear epitope devoid of tertiary structure due to the harsh denaturing that occurs during fixation and the often incomplete antigen retrieval process. As such, antibodies for IHC applications are most often selected based on their ability to recognize antigen on reduced western blots. Several of the current mAbs tested here, in particular clone 26B3, do not appear to fit this model and are therefore rather unique. This was demonstrated using FFPE sections of various normal and malignant tissues including papillary serous ovarian cancer for which FRA expression has been highly correlated independent of the type of immunological method or reagent used. Parallel analysis of the antibodies developed here demonstrated that the 26B3 antibody had the most robust binding to FRA positive tissues using either FFPE or FF preparations while tissues previously confirmed to be negative for FRA by IHC using other monoclonal and polyclonal antibodies or mRNA analysis were negative (Figure 3).

These mAbs were next tested on normal tissues from organs previously found to express FRA on the luminal surface (kidney, salivary gland, alveolar surface of the lung, etc.) and negative for FRA (ovary, liver, heart, etc.) as well as previously reported FRA-positive carcinomas (ovarian, NSCLC, renal, breast, etc.). All of the antibodies were found to show concordant staining in the positive or negative tissues as expected. Interestingly, the 26B3 antibody recognizes FRA by FACS and western blot under non-reduced conditions but not under reduced conditions. Yet, when used for IHC, the antibody is able to recognize FRA in FFPE tissues as shown in Figure 3. Previous studies using other anti-FRA antibodies such as LK26, Mov18 and Mov19 have found that these antibodies can recognize FRA in tissues prepared by FF methods only and not on FFPE tissue sections (30, authors, unpublished observations). This may be partly due to the antigen structure and its stability using various tissue fixation techniques. However, FRA stability to fixation cannot be the sole explanation since only the 9F3 mAb, which had similar binding properties to 26B3 in FACS and western blots did not recognize FRA in FFPE. One potential reason for the robust binding of mAb 26B3 to FRA in FFPE tissues is likely due to its high affinity. As noted previously, the mAbs described herein generated using the dFRA were of very high affinity (see Table 3), however, the 26B3 antibody had a distinctly slower off rate relative to all of the other mAbs tested which may account for its more robust binding. Regardless of reason, the reproducible ability to recognize FRA using different diagnostic platforms (i.e., EIA, FACS, WB, IHC on FFPE or FF tissues, etc) makes mAb 26B3 an ideal candidate for further assay development work.

**Table 3:** Affinity Constants of Selected mAbs

Antibody	k_a_ (1/M*s)	k_d_ (1/s)	K_D_ (M)
**26B3**	5.24x10^5^	1.43x10^−5^	2.73x10^−11^
**24F12**	3.93x10^5^	3.99x10^−5^	1.02x10^−10^
**19D4**	4.27x10^5^	2.42x10^−4^	5.67x10^−10^
**9F3**	4.34x10^5^	3.10x10^−4^	7.15x10^−10^

Affinities were determined using surface plasmon resonance on a BIAcore T100 instrument

To further characterize the 26B3 and a subset of other mAbs derived here, epitope binning studies were conducted to more thoroughly identify and confirm those that cross compete for FRA binding. This analysis may also be useful for gaining additional information on the three dimensional FRA protein itself using methods as previously described [31]. Two mAbs, 26B3 and 24F12, which have high affinities to purified rFRA, competed for binding to FRA, while the other antibodies appeared to bind distinct and non-overlapping epitopes. mAb 19D4 appeared to compete with LK26/MORAb-003 for FRA binding suggesting an overlap of common epitopes and/or perturbation of the three dimensional structure of FRA. The epitope binning data was confirmed by competitive FACS analysis as described above. To further refine the binding epitopes of these mAbs as well as the humanized LK26 isoform (MORAb-003) and 9F3 mAbs, H/D exchange assays were conducted.

**Figure 4:**
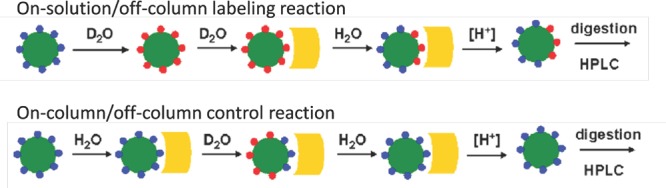
Design of H/D mapping strategy To map regions of the free FRA protein or protein bound to mAb, the protein is incubated with “on solution” that exchanges external hydrogens (depicted by blue circles) with deuterium ions (red circles). Upon saturation, the deuterated FRA is then incubated in H_2_O which exchanges hydrogen for deuterium in unbound regions or regions no longer protected by subsequent mAb binding. As control, “on-column” reactions are conducted whereby non-deuterated FRA is bound by mAb and then deuterated followed by hydrogen exchange to identify protected region bound by the anti-FRA mAb. To identify external regions on the free FRA protein, reactions are conducted with FRA at varying time points. The green circles represent the FRA protein. The yellow cylinder represents the anti-FRA antibody binding region which inhibits the hydrogen or deuterium exchange on the deuterated or non-deuterated FRA protein, respectively.

H/D-exchange analyses of rFRA alone or rFRA bound with either 9F3, 24F12, 26B3 or MORAb-003 mAbs were conducted as previously described [32]. Figure 4 provides a summary of the H/D exchange process. After H/D exchange, the external domains of the folded FRA protein as well as epitope maps for each of the antibodies were determined. The epitope for MORAb-003 was determined to be located between residues 45 and 57 (Figure 6A). This epitope was further refined using alanine-scanning mutagenesis that demonstrated changes in amino acids Ala-His-Lys-Asp within this region inhibited MORAb-003 binding to FRA (Figure 6B). Because MORAb-003 binding is limited to non-reduced FRA and competition experiments using linear or cyclized synthetic peptide corresponding to the region 45-57 failed to compete MORAb-003 binding, even at very high concentrations, these data suggest that there are additional structural determinants to MORAb-003 binding other than the linear sequence. Using similar methods, H/D exchange mapping was conducted for several of the mAbs developed in the present work. The epitope region for 9F3 was determined to be located between amino acids 17-29. Interestingly, while antibodies 26B3 and 24F12 appeared to compete with each other for FRA binding, their epitopes were located at distinct regions on the FRA protein. H/D mapping found their epitopes to be located between regions 174-185 and 67-82, respectively. This finding suggested that while the epitopes were a minimum of 92 amino acids apart on the linear protein sequence, in the naturally folded protein they appear to be in sufficiently close proximity to effect direct or indirect competition for FRA binding. A summary of the epitopes is provided in Table 4.

**Figure 5:**
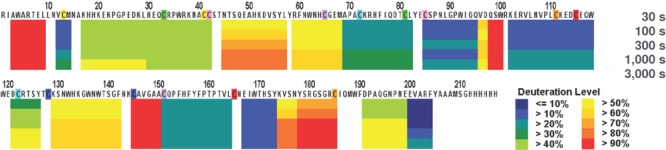
Hydrogen/deuterium exchange map of the recombinant human folate receptor alpha (rFRA) in solution H/D exchange map of rFRA at 23°C at pH7.0. Each block represents overlapping peptides analyzed. Each block contains 5 time points, as indicated on the legend to the right. Deuteration level at each time point on each peptide is indicated on the legend as well. For externally exposed regions fast H/D rates at the 30s time point that increase linearly with extended incubation periods indicate more externally exposed surfaces [32]. As shown here, amino acid regions 45-57, 129-142 and 174-184 indicate the most externally accessible regions on the FRA molecule.

**Figure 6:**
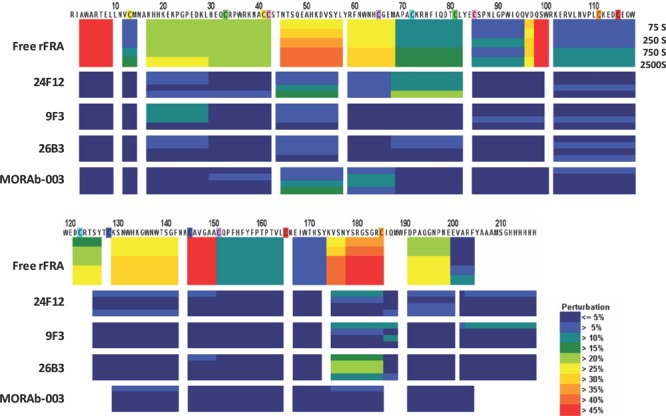

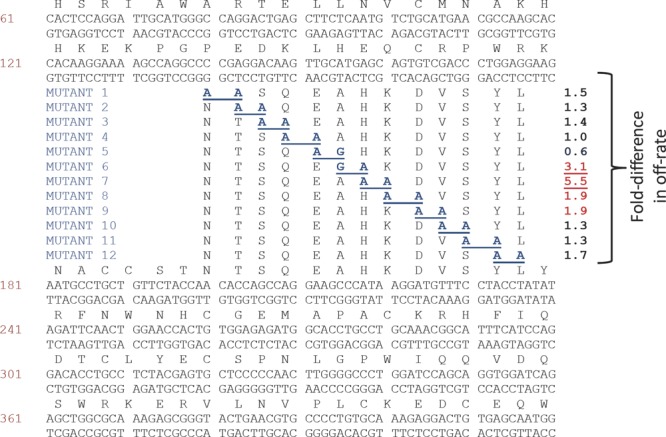
H/D mapping of anti-FRA mAb epitopes via hydrogen/deuteration exchange of FRA upon antibody complexation Shown in Panel A are the perturbations of H/D exchange of rFRA upon antibody binding, illustrated as a heat map for each antibody. The level of perturbation, as a percentage, is shown in the legend in the lower right. Included above is the H/D exchange map of free rFRA in solution for comparison and alignment of the proposed epitope regions of 24F12, 9F3, 26B3 and MORAb-003 mAbs, respectively. For MORAb-003, epitopes were confirmed via di-alanine scanning of full length rFRA vs mutant FRA variants which refined the epitope for the antibody from the 45-57 region to amino acid residues HKDV (Panel B). The H/D mapped epitope regions for each mAb is identified and provided in Table 4. **Panel A.** H/D exchange map of anti-FRA mAbs on rFRA at varying incubation time points. The antibody for each exchange map is shown on the left and the amino acid sequence of FRA is shown on the top. **Panel B.** Di-alanine scanning of FRA of the MORAb-003 epitope region 45-57 identified via H/D mapping was conducted to further refine the epitope of the antibody. MORAb-003 had reduced binding of FRAs containing changes within the HKDV amino acids as determined via surface plasmon resonance. Shown are the 12 mutant di-alanine full length FRA mutants and the binding characteristics of MORAb-003 to each mutant with values shown on the right.

**Table 4:** Epitope Regions of FRA Specific mAbs Identified by H/D Mapping

Antibody	Epitope Region on FRA Protein (amino acid positions)
26B3	174-185
24F12	67-82
9F3	17-29
MORAb-003	45-57

The reagents generated here and methods by which they were made provide valuable tools for detecting FRA protein in tissues and cells prepared using various standard methods employed by pathology labs as well as techniques such as EIA. Moreover, as we attempt to learn more about the structure and function of how FRA participates in cellular transformation, a subset of these reagents may be useful in providing more information on the three dimensional structure of the protein until the crystal structure of the protein and other three dimensional modeling studies are successfully conducted. Of particular interest are the two antibodies generated against native FRA, MORAb-003 and 9F3 which are only able to detect non-reduced FRA protein as well as FRA in tissues prepared only under FF methods, not in FFPE prepared samples. Additionally, the 26B3, 24F12 and 19D4 mAbs provide interesting reagents in that they are able to recognize FRA under non-reduced conditions but in tissues prepared via FFPE as well as FF, again distinguishing the interesting conformation of the epitopes to which they bind. These reagents have also been demonstrated to be effective in measuring FRA in biological fluids such as serum and plasma (authors, unpublished data).

## DISCUSSION

The primary aim of the present study was to develop new reagents that could reproducibly detect FRA expression, primarily on tissues or in bodily fluids derived from cancer patients in an attempt to generate reagents suitable for FRA diagnostics and to support FRA-based therapies. These efforts, if successful, could potentially enable the development of a diagnostic assay that could serve as a suitable probe for staging, differentiating and/or evaluating the antigen in cancer for prognosis. While other anti-FRA reagents have been previously developed, a high degree of variability in FRA expression profiles across various cancers has been observed likely due to the platform and methods of sample preparation [7, 29, 30]. Here we describe the development of a panel of distinct, high affinity mAbs that recognize different regions of the FRA protein. Of particular note is the development of mAb 26B3 which is able to detect FRA using a number of standard sample isolation and detection platforms. The robustness of this antibody is likely due to its high affinity that is driven by its slow dissociation constant and the unique epitope to which it binds. While many of the antibodies developed here, as well as in previous studies, have been shown to recognize native specific structures, none have been able to detect FRA in tissues prepared by various platforms that include native and denaturing fixation methods. Interestingly, the 26B3 mAb is able to recognize FRA in its native state and denatured form likely due to its recognition of an epitope that juxtaposes a cysteine residue that retains significant structure of the FRA protein in samples prepared using a variety of tissue fixation methods. This hypothesis is further supported by the finding that the antibody is able to detect FRA in non-reduced but not in reduced western blots (Figure 2). This antibody is now being tested in broad cancer association studies in parallel with other previously reported antibodies [33, 34, 35] to compare its usefulness as a diagnostic reagent to support patient screening for FRA-based targeted therapies as well as its use for identifying, staging and/or predicting clinical outcomes of patients with FRA positive cancers.

**Figure 7:**
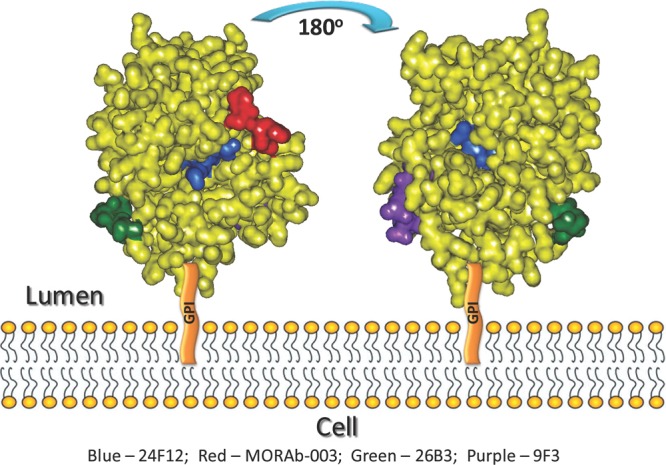
Modeling of the anti-FRA antibody epitopes on the FRA-related structure, chicken riboflavin-binding protein (cRBP) Sequences of cRBP and hFRA were aligned and linear sequences of anti-FRA antibody epitopes as well as external domains of the free FRA protein were mapped on cRBP and highlighted on the cRBP 3-D structure. The model is shown from two different angles to depict the epitopes for each antibody as well as the free protein analyzed by H/D mapping. As anticipated by the H/D method, those regions showing highest H/D exchange are localized to the external regions of the FRA protein.

Another finding from this study has been the use of the panel of mAbs and H/D exchange mapping of the FRA protein itself or when bound by one of the mAbs developed here to further define the three dimensional structure of the FRA protein. The unique binding properties of these mAbs have enabled us to evaluate regional domains of the FRA protein as they relate to their positioning on the external surface. As the FRA protein is a highly compact multi-disulfide linked structure, the positioning of its residues on the external regions of the protein in the native state has been better defined by analyzing the specific binding of the mAb panel to the FRA protein in the native and denatured state, followed by their epitope mapping. Indeed those residues predicted to be present on the exterior of the protein and confirmed via H/D mapping of the free FRA protein are recognized by mAbs that bind preferentially to the native (LK26, 19D4 and 9F3) but not denatured forms of the protein. Evaluating the binding characteristics and epitopes of various mAbs generated here along with H/D exchange mapping of the native protein can identify domains that are freely exposed to the surface of the molecule and potentially provide additional information on the three dimensional structure of the protein. As shown in Figure 5, the regions within amino acids 96-100, 145-150, and 174-185 appear to be freely exposed based on the H/D exchange mapping of the free protein. The regions 45-57 and 129-142 appear also to be accessible to external exposure albeit to a lesser extent. These findings corroborate the predicted three dimensional structure of FRA generated by Shen et.al. who used molecular modeling of FRA based on the conserved chicken riboflavin (cRBP) protein [36]. Figure 7 shows a highlight of the external surfaces predicted by H/D exchange extrapolated onto the cRBP 3-dimentional model. These results showed a good correlation between the predicted external domains and those identified by the H/D exchange mapping. The additional binding characteristics of the lead antibodies developed here have further supported the external topography of these regions based on the ability of the mAbs to recognize FRA under reduced and/or non-reduced conditions. The overlay of the epitope regions for each mAb on this model enables the three dimensional visualization on how these regions juxtapose each other and how binding of these mAbs to FRA may effect their ability to bind or inhibit binding of various mAb pairs on the native protein.

The data presented here describe the development of new antibodies that are able to detect FRA using a variety of methods. These antibodies and the models used for mapping provide additional information about the FRA protein and how it may present itself on the cell surface [35, 36]. These data are important for further defining regions that may be accessible to new targeting entities (i.e. antibodies, antibody fragments, peptides, etc.) that can deliver novel pharmacologic agents to FRA-positive cells. Moreover, recent findings by Knutson et.al [37] have found that the FRA protein has external regions that may be recognized by the patient's immune system in those exhibiting various FRA-positive malignancies, including breast and ovarian cancer. The epitopes identified by autoimmune responses may further aid to provide additional information on the native structure of the protein as well as how FRA may interact with other proteins located in the lipid membrane raft as it signals to cells for enhanced or sustained growth [18, 38]. The antibodies described herein are now being employed in large scale analysis of epithelial-based cancers to determine if FRA is a useful marker for detecting, staging and or determining the prognosis of patients with FRA positive cancers.

## MATERIALS AND METHODS

### Expression and purification of Sf9-expressed human FRA

A human folate receptor alpha (FRA) sequence, containing a leader sequence optimized for Sf9 bacculovirus expression, an N-terminal histidine (6xhis) epitope tag, and the intact native GPI attachment site, was used to prepare a high-titer bacculovirus vector capable of expressing the recombinant human FRA. 1L shake flask cultures containing log-phase Sf9 insect cells were infected with the recombinant bacculovirus at a multiplicity of infection (MOI) of <1. Cells from 30L of culture were harvested and lysed/extracted twice with 1X PBS containing 10mM CHAPS. The NaCl concentration was adjusted to 300mM and filtered through a 0.2μm membrane. The clarified supernatant was purified via anti-FRA affinity chromatography, using 1X PBS with 2M NaCl, 1mM CHAPS, pH7.4 as wash buffer, followed by elution with 10mM MOPS, 3M MgCl_2_, 1mM CHAPS, pH6.8. Peak fractions were dialyzed extensively against 1X PBS, pH7.4, analyzed for purity by SDS-PAGE, quantitated by bicinchoninic acid (BCA) assay, aliquotted and stored at -80^o^C.

### Expression and purification of soluble human FRA from recombinant CHO cells

A human FRA sequence, containing a human immunoglobin kappa leader sequence and a C-terminal 6xhis epitope tag replacing the GPI attachment site, was used to prepare a Chinese hamster ovary (CHO) cell line stably expressing and secreting human FRA as described [39]. Transfected cells were grown in 25L wave bags. Supernatants were collected, filtered to remove debris, concentrated 10-fold by tangential flow filtration, and then diafiltered into 50mM sodium phosphate, 300mM NaCl, 1mM imidazole, pH8.0. Samples were then loaded onto a pre-packed Talon IMAC column and fractionated using FPLC. Protein fractions were eluted using a linear gradient of 5-100mM imidazole in 50mM sodium phosphate, 300mM NaCl, pH8.0. Peak fractions were dialyzed against 1X PBS, pH7.4, analyzed for purity by SDS-PAGE, quantitated by BCA assay, aliquotted and stored at -80^o^C.

### Expression and purification of soluble human FRA, FRB, FRD, and FRG from transiently-transfected 293F cells

Recombinant vectors containing full length cDNAs encoding for human FRA, FRB, FRD, and FRG were all engineered to contain a human immunoglobulin kappa leader sequence and a C-terminal 6xhis epitope tag that replaced the GPI attachment site. Vectors were then used to transiently transfect 1L cultures of human 293F cells as previously described [39]. After 10 days of culture, cells were harvested and debris was removed by centrifugation. Supernatants containing secreted FR proteins were diafiltered and purified as described above.

### Immunogen preparation

Purified recombinant FRA (rFRA) derived from Sf9 cells was concentrated to 2mg/mL in PBS (pH7.4) using centrifugal filters (Amicon Ultra, 3kDa MWCO) and quantified using a BCA assay according to the manufacturer (Thermo). To generate reduced/alkylated, denatured FRA (dFRA), a portion of the rFRA preparation was diluted 1:1 in 8M urea/PBS to generate a final concentration of 1mg/mL FRA in PBS containing 4M urea. Dithiothreitol (DTT) solution (500mM in PBS) was added to a final concentration of 10mM. The solution was incubated at 65^o^C for 1hr, and cooled to room temperature (RT). Next, 1M iodoacetamide solution in PBS was added into the reduced folate receptor solution to a final concentration of 10mM, and the reaction was kept in the dark at RT for 30min. The final reduced/alkylated FRA was dialyzed into PBS containing 4M urea, 10mM DTT and 10mM iodoacetamide and stored at -80^o^C until use.

### Immunization, cell fusion and hybridoma cloning

Five eight-week old female Balb/c mice were immunized with rFRA or dFRA protein as follows. Initial intraperitoneal immunizations administered on day 0 comprised 50μg of rFRA or dFRA prepared as a 1:1 (v:v) mix with complete Freund's adjuvant (Rockland Immunochemicals, Inc., Gilbertsville, PA). Boosts (50μg immunogen mixed 1:1 (v:v) with incomplete Freund's adjuvant; Rockland Immunochemicals Inc.) were administered intraperitoneally on day 14 and every 21 days thereafter. Test bleeds were collected on day 24 and every 21 days thereafter and analyzed by direct enzyme-linked immunoassay (EIA) against rFRA (see below). Spleens were harvested from animals exhibiting the highest antigen-specific titers and hybridomas were prepared by electrofusion (Hybrimune™ Model CEEF-50B Waveform Generator; Cellectis, Romainville, France) of splenocytes with Sp2/0 Ag14 myeloma cells (ATTC, Rockville, MD). Hybridoma supernatants were rescreened by EIA against rFRA or recombinant mesothelin protein as a negative control, to identify hybridomas producing anti-FRA antibodies. Selected parental cell lines were then sub-cloned by limiting dilution and re-assayed against rFRA by EIA. Isotyping of selected clones was performed using the Clonetyping System (SouthernBiotech, Birmingham, AL).

### Monoclonal antibody (mAb) selection – Direct EIA

mAb selection was performed using a direct EIA technique. Briefly, 96-well plates were coated with 100μL/well of a PBS solution containing 0.5μg/mL rFRA overnight at 4^o^C. Wells were then washed and blocked with 3% (w/v) fish gel in PBS for 1hr at RT. Next, 100μL of a 3-fold dilution series of hybridoma culture supernatant was added to the wells and plates were incubated at RT for 1hr. After washing 3 times with PBS containing 0.2% (v/v) Tween-20 (PBST), 100μL of horseradish peroxidase-conjugated rabbit anti-mouse IgG (1:2500 dilution; Rockland Immunochemicals, Inc.) was added and plates were incubated for 30min at 37^o^C. After washing with PBST, 100μL of substrate (TMBE-100; Rockland Immunochemicals, Inc.) was added and the reaction stopped after 30min by addition of 100μL of 1M HCl. Plates were read at 450nm on a Benchmark micro-plate reader (BioRad, Concord, CA).

### mAb Selection – Immunohistochemistry (IHC) on FFPE tissue secondary screen

Hybridoma cell culture supernatants were screened by IHC (see below) on 5μm formalin fixed paraffin embedded (FFPE) sections of papillary serous ovarian carcinoma. Preliminary positivity in IHC was used to aid selection of hybridomas for further analysis and optimization in IHC.

### mAb production and purification

Hybridoma cell lines were tested for mycoplasma using the mycoplasma PCR ELISA (Roche, Mannheim, Germany). mAbs were produced from mycoplasma negative cells grown in 1L roller bottle cultures. After seeding at 0.5x10^5^ cells/mL, cultures were grown for 14 days in serum free medium (Invitrogen) and 5% low IgG fetal bovine serum (Gibco). At termination, culture supernatants were concentrated approximately 10-fold through a 50kDa filtration membrane (SpectrumLabs, Rancho Dominguez, CA) and mAbs were purified by Protein A affinity chromatography, eluted with 0.1M sodium citrate pH3.5 buffer, and subsequently dialyzed against PBS using a 12-14kDa membranous tubing (SpectrumLabs, Rancho Dominguez CA), sterile filtered using 0.22μm Express™PLUS Stericups (Millipore, Billerica MA), aliquotted and stored at -80^o^C.

### Fluorescense Activated Cell Sorting (FACS) analysis

The specificity of the anti-human FRA mAbs was assessed by FACS analysis using stably transfected CHO cell lines, each expressing a different member of the folate receptor family. cDNAs encoding the full length FRA, FRB or FRD homologs were cloned into a eukaryotic expression vector and transfected into CHO cells and selected for expression as previously described [39]. After establishment of stable FRA, FRB and FRD expressing CHO cells, cells were expanded and tested for FRA, FRB, and FRD expression by RNA and western blot using polyclonal antisera generated against each antigen. Stable cell lines were then used to study FR expression and as reagents to screen for FRA specific antibodies. For FACS, cells were harvested in PBS/EDTA, washed, and re-suspended in ice-cold growth media (RPMI supplemented with 10% FBS). Cells were incubated for 1hr on ice with 1μg/mL of a mAb, washed and then incubated with FITC-conjugated secondary antibodies [dilution 1:100] (Southern Biotech, Birmingham, AL). Prior to analysis, cells were labeled with 7-Amino-actinomycin D (7-AAD) (BD Biosciences, Franklin Lakes, NJ) for the exclusion of nonviable cells. Cells were analyzed on an EasyCyte Flow Cytometer (Guava Technologies, Hayward, CA). Data is presented as graphical representation of mean fluorescence intensity (MFI) of 7-AAD negative, viable cells. For FACS competition studies, cells were first incubated with one antibody and then washed and re-incubated similarly with a second antibody and then analyzed on the EasyFlow Cytometer as described above.

### Western Blot Analysis of Cell Lysates

CHOK1 transfected cells stably expressing GPI-linked human FRA, FRB or FRD respectively were lysed in 1.1% OBG buffer (50mM Tris-HCl, pH7.5, 150mM NaCl, 1.1% OBG) supplemented with Complete Mini Protease Inhibitor Cocktail (Roche Diagnostics, Indianapolis, IN) and PMSF (100nM), and placed on ice for 15min. Lysates were pre-cleared by centrifugation at 13,000rpm for 15min to remove debris. Equal amounts of protein (20μg) were boiled for 10 min in NuPAGE LDS sample buffer (Invitrogen) with 5% β-mercaptoethanol (BME) and 40mM DTT for reducing gels or without for non-reduced gels. Proteins were separated using SDS-polyacrylamide gel electrophoresis (SDS-PAGE) on a 4-12% Bis-Tris gel (Invitrogen) and electroblotted onto PVDF membrane. After transfer, the membrane was blocked in PBST and 5% non-fat milk for 1h at RT and then washed twice with PBST. Immunoblotting was conducted using purified anti-FRA mouse mAbs (1μg/mL), detected with a goat-anti-mouse HRP-conjugated antibody and visualized using SuperSignal West Pico chemiluminescent substrate (Pierce, Rockford, IL). The LK26 antibody was used as a positive control for FRA detection. Luminescence was visualized using the Omega 12iC molecular imaging system (Ultra-Lum, Claremont, CA) with image analysis performed using UltraQuant 6.0 software (Ultra-Lum).

### Western blotting using purified FR isoforms

0.5μg of purified human FRA, FRB, FRD, and FRG produced as described above were incubated in 1X SDS-PAGE sample loading buffer (Invitrogen) with or without 20mM DTT, boiled for 10min, and electrophoresed on 4-12% gradient SDS-PAGE gels. Protein was transferred to PVDF membrane and blots probed as described above. Gels were run using Benchmark™ prestained protein ladder (Novex^®^). Gels using purified recombinant FR proteins were also visualized via silverstaining to ensure equal amounts of protein were loaded.

### Immunohistochemistry

Immunohistochemical (IHC) testing to detect FRA was performed using formalin fixed paraffin embedded (FFPE) or fresh frozen (FF) tissues. For FFPE specimens, IHC was performed using a MACH4 Universal HRP-Polymer Detection Kit (Biocare Medical, Concord, CA). FFPE specimens were sectioned at 5μm on positively-charged glass slides and heated for approximately 60min at 60°C. Slides were deparaffinized in 3 sequential baths of xylene for 3min each, transferred to three sequential baths of 100% alcohol for 3min each, followed by three sequential baths of 95% alcohol for 3min each and then rinsed for 5min in deionized (DI) water. Slides were then pretreated in Diva heat-induced epitope retrieval solution (Biocare Medical) diluted 1:10 in DI water and placed inside a pressurized decloaking chamber already filled with 500mL of DI water. For antigen retrieval, slides were incubated for 15min inside the decloaking chamber in which pressurized incubation reaches a maximum of 125C at 16 PSI for 30sec and then cooled for 15min down to 95°C. After cooling to RT, slides were washed in 3 sequential baths of Tris Buffered Saline/0.1% Tween-20 wash buffer (TBST) for 3min each and subsequently placed into Peroxidase-1 (Biocare Medical) blocking solution for 5min at RT. After washing in TBST as above, Background Sniper (Biocare Medical) serum-free universal blocking reagent was applied for 10min at RT. Slides were then incubated with either individual anti-FRA cell culture supernatants (hybridoma secondary screening; undiluted culture supernatant) or purified mAbs at 2.5μg/mL diluted in Antibody Diluent (Dako) or Universal Negative Control [mouse ready-to-use negative control antibody (Dako, for negative isotype tissue)] for 60min at RT. After washing, slides were incubated with MACH4 Mouse Probe Primary Antibody Enhancer for 15min, followed by Polymer-HRP reagent for 20min, developed with a 3,3′-diaminobenzidine tetrahydrochloride (DAB) solution (Dako) for 5min and counterstained with hematoxylin (Dako) for 2min, all incubations being performed at RT. For IHC on fresh frozen (FF) sections, tissues were harvested and snap frozen in liquid nitrogen and stored at -80°C until used. FF tissue samples were sectioned at 5μm onto positively-charged glass slides at 4°C and subsequently stained using the methods described above, excluding the deparaffinization steps.

### Surface Plasmon Resonance

All surface plasmon resonance (SPR) experiments were performed at 25°C using a BIAcore T100 with research grade CM5 chips (GE Healthcare). Following the instructions provided by the manufacturer, anti-mouse IgG provided in the mouse antibody capture kit (GE Healthcare) was immobilized by amide coupling to CM5 sensor chips. Murine anti-human FRA mAbs were captured on individual flow cells per binding cycle, while the fourth flow cell was used as a reference. Binding experiments were performed with HBS-P (GE Healthcare) as running buffer and at a flow rate of 30μL/min. Each mAb sample (0.5μg/mL) was injected for 3min to capture the antibody. Various concentrations of purified rhFRA (1nM - 30nM) were then injected over the anti-FRA flow cells and reference surfaces for 3min to record binding sensograms using a single-cycle kinetics method. The dissociation profile was monitored for 25min. In between bindings, the surface was regenerated with a 30μl injection of 10mM glycine (pH1.7). The sensograms were processed and fitted to a 1:1 Langmuir binding model using BIAcore T100 evaluation software (version 2.0.1).

### Epitope binning by biolayer inferometry

Antibody epitopes were analyzed to localize FRA binding by first epitope binning techniques [31] and subsequently further refined using hydrogen-deuterium (H/D) exchange mapping (below) [32]. Epitope binning was performed at 30°C using an Octet QK (ForteBio) with PBS containing 0.005% Tween20 (PBST) as buffer. Purified human rFRA was biotinylated with EZ-Link Sulfo-NHS-LC-Biotin following the protocol provided by the manufacturer (Thermo Scientific). Briefly, a 20-fold molar excess of biotin was incubated with rFRA for 40min at room temperature. The biotinylated rFRA was then dialyzed extensively against PBS at 4°C. Streptavidin-coated biosensors (SA sensors, ForteBio) were pre-wet for 10min in PBST containing 0.1% BSA, immediately before use. Biotinylated rFRA was coupled onto SA sensors by dipping the sensors in microplate wells containing 220μl of the biotinylated FRA (3μg/ml) for 10min at an agitation speed of 300rpm. A new baseline was established for each sensor in PBST buffer for 3min. The FRA-coupled biosensors were dipped into a column of wells containing a primary saturating anti-FRA mAb (400nM) for 10min at an agitation speed of 1500rpm. Then the biosensors were moved into a column of wells containing six different secondary competing anti-FRA mAbs (400nM, 10min). Two wells containing PBST and the primary anti-FRA mAb, respectively, were included in the column for referencing purposes. The dissociation of anti-FRA mAbs was monitored for 5min.

### Epitope mapping by hydrogen/deuterium exchange

#### Immobilization of antibody

Purified monoclonal antibody was immobilized on POROS 20 AL media (Applied Biosystems) per the manufacturer's instructions. Briefly, 100mgs of POROSAL and 4mg sodium cyanoborohydride (NaCNBH_3_) were added to 1mg of mAb. After addition of 0.5mL 2.8M sodium sulfate (Na_2_SO_4_), the reaction mixture was shaken at RT overnight. The resin was washed with PBS and then shaken with 2mL of PBS containing 1M ethanolamine and 8mg/mL of NaCNBH_3_ at RT for 2h to cap the unreacted aldehyde groups. The capped resin was washed with PBS and resuspended in PBS.

#### On-Exchange of rFRA in Solution

In H/D exchange studies, “on” reactions refer to those in which protein(s) are exposed to deuterium solutions, resulting in the exchange of hydrogen for deuterium. “Off” reactions, conversely, refer to those in which deuterated proteins are exposed to non-deuterated solutions, resulting in the exchange of deuterium with hydrogen. On-exchange experiments with human rFRA were performed in order to assess the dynamic properties of the protein in solution and identify regions of fast exchange (solvent-exposed) and slow-exchange (interior regions). ‘On-solution’ (on-exchange in solution) reactions were initiated by diluting 5μL of 1mg/mL rFRA with 35μL of deuterated buffer (1X PBS, 87% D_2_O). The reaction mixture was incubated at 23°C for varying times (30, 100, 300, 1000, and 3000s). Fully deuterated sample was prepared by mixing 5μL of a 1mg/mL rFRA with 35μL of 100mM TCEP in D_2_O and incubating at 60°C for 3h. 20μL of 2M urea, 1M TCEP, pH3.0 was added to the exchange reactions to quench the deuteration. The mixture was subjected to papain digestion, LC-MS/MS, and data analysis (see below).

#### Principle of epitope mapping by H/D exchange using immobilized monoclonal antibody

In the labeling experiment, rFRA is on-exchanged in solution for a predetermined time and then off-exchanged in the antibody column for the same duration (Figure 4). The anti-FRA epitope becomes deuterated during the on-exchange as it is exposed to the solvent and may retain deuterium during the off-exchange as it may be protected by the anti-FRA antibody. Conversely, regions that are not affected by antibody binding may be deuterated during the on-exchange but will lose deuterium during the off-exchange. More information about H/D epitope mapping can be found at www.exsar.com.

#### ‘On-solution/off-column’ analysis

‘On-solution’ (on-exchange in solution) reactions were initiated by diluting 5μL of 1mg/mL rFRA with 35μL of deuterated buffer (1X PBS, 87% D_2_O). The reaction mixture was incubated at 3°C for varying times (150, 500, 1500, and 5000s). The on-exchanged solution was loaded onto the antibody column that was pre-equilibrated with deuterated buffer, and then washed once with deuterated buffer. ‘Off-column’ (off-exchange in column) reactions were initiated by washing the column with 200μL of chilled 1X PBS. ‘Off-column’ reactions continued for one half of the preceding on-exchange duration (75, 250, 750, and 2500s). The introduction of 140μL of 0.8% formic acid quenched the exchange reactions and eluted out the FRA from the antibody column. The last 40μL of the eluent was collected and added to 20μL of 2M GuHCl, 1M TCEP, pH 3.0. The mixture was subjected to papain digestion, LC-MS/MS, and data analysis (see below).

#### ‘On-column/off-column’ analysis

The column from above was regenerated by washing twice with 250μL 0.8% formic acid, then equilibrated twice with 250μL of ice chilled PBS. 5μL of 1mg/mL rFRA was diluted with 35μL of ice chilled 1X PBS (0% D_2_O). The reaction mixture was incubated at 3°C for varying times (150, 500, 1500, and 5000s). The solution was loaded onto the antibody column and then washed once with 1X PBS. ‘On-column’ reactions were initiated by washing the column with 100μL of ice chilled deuterated buffer (1X PBS, 87% D_2_O). ‘On-column’ reactions continued for 75, 250, 750, and 2500s. Subsequent off-column, elution, papain digestion, LC-MS/MS, and data analysis were identical to on-solution/off-column analysis.

#### Papain digestion, LC-MS/MS, and data analysis

For papain digestion, samples were passed over an immobilized pepsin column at 200 μL/min in buffer A (0.05% TFA in H_2_O). Peptic fragments were loaded onto a reversed-phase trap column and desalted with buffer A at 200μL/min for 3min. Peptic peptides were separated by a C18 column with a linear gradient of 13% to 40% buffer B (95% acetonitrile, 5% H_2_O, 0.0025% TFA) in buffer A for 23min. Peptic peptides were analyzed by mass spectrometry using a Thermo Finnigan LCQ^TM^ mass spectrometer (Thermo Fisher, San Jose, CA) with the capillary temperature set at 175°C in MS1:Profile mode. Deuteration level of each peptide analyzed was analyzed as described in Coales et al [32].

### Refinement of MORAb-003 epitope by alanine scanning mutagenesis

Mutant soluble human FRA constructs were designed such that adjacent pairs of amino acids in the MORAb-003-protected region of FRA identified by H/D exchange mapping (amino acids 45-57) were mutated to alanine, with the exception of Ala50, which was changed to glycine. Mutant FRA constructs overlapped by one amino acid, generating a total of 12 mutant FRA constructs. These mutant FRA constructs, along with wild-type FRA, were transiently-transfected into 293F cells and supernatant was collected, centrifuged, and filtered. For BIAcore analysis of mutants, a CM5 chip was prepared having 12,000 RU PentaHis anti-his tag antibody immobilized on all four flow cells (Qiagen) using standard EDC/NHS chemistry on a BIAcore 3000 instrument. Wild-type and mutant FRA supernatants were diluted 1:10 in HBS-EP buffer and were captured on flow cells 2-4 of the PentaHis chip at RU levels of 100-200 RU. Flow cell 1 was used as a reference flow cell. MORAb-003 (0nM-30nM in HBS-EP buffer) was injected over all four flow cells. Data was double-referenced, and kinetic constants were determined using BIAevaluations.
